# Changes in the Management of Common Bile Duct Stones: 1980 to Date

**DOI:** 10.5041/RMMJ.10521

**Published:** 2024-04-28

**Authors:** Ruth Stalnikowicz, Jochanan Benbassat

**Affiliations:** Department of Medicine (retired), Hadassah University Hospital, Jerusalem, Israel

**Keywords:** Common bile duct calculi, controlled trials, choledocholithiasis

## Abstract

**Objective:**

To compare the results of treating patients with common bile duct (CBD) stones by endoscopic sphincterotomy (ES), surgical exploration, or a combination of ES and surgical CBD exploration (the rendezvous technique).

**Methods:**

A narrative review of the literature.

**Summary of Data:**

Before 1990, 17 cohort studies indicated that ES cleared CBD stones in 92.0% of patients, with a mortality rate of 1.5%. Surgery removed CBD stones in 90.2% of patients, with a 2.1% mortality rate. A single randomized controlled trial in 1987 showed that ES removed CBD stones in 91% of 55 patients, with a 3.6% mortality rate and a 27% complication rate, whereas surgical CBD exploration removed CBD stones in 92%, with a 1.8% mortality rate and a 22% complication rate. Since 1991, 26 randomized controlled trials have shown that laparoscopic–ES rendezvous is as effective as ES alone and laparoscopic surgery alone but is associated with fewer complications, a reduced need for additional procedures, and a shorter hospital stay.

**Conclusions:**

A laparoscopic–ES rendezvous appears to be the optimal approach to the treatment of CBD stones in younger and fit patients. The choice between ES alone and laparoscopic–ES rendezvous in older or high-risk patients remains uncertain.

## INTRODUCTION

The alternative approaches to the management of patients with common bile duct (CBD) stones include open surgical choledochotomy, endoscopic sphincterotomy, and laparoscopic CBD exploration. Open choledochotomy requires anesthesia and abdominal surgery. Endoscopic sphincterotomy (ES) entails upper endoscopy, identification of the ampulla of Vater, and clearance of the CBD stones after sphincterotomy. Since it was described in 1974, ES is an established technique for CBD stone removal.^1^ Laparoscopic CBD exploration was first employed in the 1990s. It is as safe and efficient as open surgery, provides a clearer vision, and is associated with less pain and quicker recovery.^2^ As many as 26% of the patients who were discharged with gallbladders *in situ* after CBD clearance required follow-up cholecystectomy for biliary symptoms.^3^ Therefore, it is considered best practice to combine CBD clearance with cholecystectomy.

Endoscopic sphincterotomy may be complicated by failure to cannulate the ampulla of Vater and pancreatitis that can follow inadvertent pancreatic cannulation and contrast injection. Hence the advantage of the *simultaneous* clearance of CBD stones by ES *during* laparoscopic surgery (laparoscopic–ES rendezvous).^4^ The laparoscopic–ES rendezvous procedure is a single-stage laparoscopic and endoscopic approach to CBD treatment that minimizes the risk of inadvertent pancreatic duct cannulation and pancreatitis, thereby leading to a shorter hospital stay, higher success rate, and lower cost. It entails *laparoscopic* incision of the cystic duct by a first team of surgeons; introduction of a radiopaque guiding catheter toward the duct; intraoperative cholangiography to confirm biliary stones; and introduction of a guidewire through the biliary tree into the duodenum under radiological guidance. This guidewire helps the *endoscopic* identification of the ampulla of Vater by a second team who also clears the CBD stones.

The choice between these alternatives depends on the severity of the symptoms, the presence of the gallbladder, and local expertise. Endoscopic sphincterotomy has gained acceptance in the treatment of patients with retained CBD stones after cholecystectomy and in high-risk patients with intact gallbladders. Its role in the treatment of younger and fit patients is uncertain. In 1980–1990, this uncertainty led to different management of CBD stones: in the United States, only 15% of the patients with intact gallbladders and biliary symptoms had ES; in Europe, this figure exceeded 60%.^5^ The choice between laparoscopic–ES rendezvous and preoperative ES is similarly uncertain. A 2018 Cochrane Review of five randomized trials concluded that there was insufficient evidence to determine this choice because the quality of evidence was low.^6^

The aim of the present study is, first, to review the changes in the treatment of patients with CBD stones since 1980; and, second, to compare the results of ES alone, surgical (open and laparoscopic) CBD exploration, and laparoscopic–ES rendezvous to determine the optimal approach for the treatment of patients with CBD stones.

## METHODS

This is not a systematic review. We searched a single database (Google Scholar) for reports published in English since 1970 on combinations of the following terms: choledocholithiasis, cholecystectomy, common bile duct calculi, randomized trials, and choledocholithiasis. Additional studies were sought by screening the bibliographies of review articles. We included uncontrolled cohort studies published before 1990 and randomized trials since 1990. We excluded reports of patients with previously attempted CBD stone clearance, malignant biliary disorders, and those that compared the timing of cholecystectomy after ES. The number of patients with CBD stones in the reported series was the denominator for calculations of the success rate in clearing CBD stones, while the total number of patients entered into the trial, including those with suspected but absent CBD stones, was the denominator for complications and mortality.

## DATA SYNTHESIS

Table 1 summarizes *uncontrolled cohort studies* published before 1990; Tables 2 and 3 present their results in more detail. Endoscopic sphincterotomy cleared CBD stones in 92.0%, with a mortality of 1.5%, complications in 8.2%, and a need for additional treatment in 3.3%. The most common complications after ES were hemorrhage and duodenal perforation after cutting the papilla, and acute pancreatitis and cholangitis after injection of the contrast media.^7^ Before 1990, open surgical CBD exploration cleared CBD stones in 90.2% of the patients. Mortality was 2.1%, and complications occurred in 18.3%. Reoperations, needed in 6.5% of the patients, were most commonly for recurrent/retained stones, stenosis of the ampulla, and pancreatitis.^8^ In both ES and surgical CBD exploration, complications and mortality increased with age.

**Table 1:** Summary of the Outcomes of Uncontrolled Cohort Studies Published Before 1990 of Endoscopic Sphincterotomy (ES) and Open Surgical Common Duct Exploration (Surg.).

Years	Intervention	Patients *n*	Stone Clearance *n* (%)*	Additional CBDI needed (ES or Surg.) *n* (%)†	Complications *n* (%)†	Mortality *n* (%)	Length of Hospital Stay d (range)
1982–1989	ES	3,818	2,210/2,403 (92.0)	110/3,306 (3.3)	312/3,798 (8.2)	56/3,818 (1.5)	3–6
1972–1990	Surg.	3,606	1,952/2,164 (90.2)	155/2,391 (6.5)	575/3,142 (18.3)	77/3,606 (2.1)	10–16

The short mean hospital stay of patients undergoing ES suggests that mortality rate and hospital stay from the subsequent cholecystectomy were not taken into account.

* The denominators include the total number of patients entered into the trial, after excluding those with suspected but absent CBD stones.

† The denominators include the number of patients with available data.

CBDI, common bile duct interventions; d, day(s).

**Table 2:** Uncontrolled Studies Published Before 1990 of the Outcomes of Endoscopic Sphincterotomy in Patients with Common Bile Duct Stones.

Author	Mean age y	Patients *n*	Stone Clearance *n* (%)*	Additional CBDI Needed (ES or Surg.) *n* (%)†	Complications *n* (%)†	Mortality *n* (%)	Length of Hospital Stay d (range)
Cotton (1982)^18^	75.0	71	61/71 (85.9)	2/71 (2.8)	2/71 (2.8)	1/71 (1.4)	NG

Danilewitz (1984)^11^	61.9	53	48/53 (90.6)	NG	5/53 (9.4)	0/53 (0.0)	NG

Escourrou et al. (1984)^21^	79.0	443	428/443 (91.2)	7/443 (1.6)	27/443 (6.1)	6/443 (1.4)	NG

Neoptolemos et al. (1984)^20^	78.0	100	91/100 (91.0)	5/100 (5.0)	13/100 (13.0)	1/100 (1.0)	NG

Roberts-Thomson (1984)^12^	63.0	300	147/164 (90.0)	5/300 (1.7)	14/300 (4.7)	0/300 (0.0)	NG

Leow and Thompson (1986)^22^	79.5	20	16/20 (80.0)	NG	NG	0/20 (0.0)	NG

Duron et al. (1987)^25^	83.0	33	25/26 (96.2)	1/33 (3.0)	0/33 (0.0)	2/33 (6.1)	NG
Neoptolemos et al. (1987)^19^	75.7	190	156/190 (82.1)	19/190 (10.0)	33/190 (17.4)	15/190 (7.9)	NG

Davidson (1988)^17^	79.0	106	98/106 (97.1)	6/106 (5.7)	21/106 (19.8)	12/106 (11.3)	NG

Ikeda et al. (1988)^13^	65.0	469	438/469 (93.4)	NG	30/469 (6.4)	2/469 (0.4)	NG

Miller et al. (1988)^14^	68.0	156	133/156 (85.3)	18/156 (11.5)	22/156 (14.1)	5/156 (3.2)	6.0

Siegel et al. (1988)^5^	73.3	1272	NG	25/1272 (2.0)	109/1272 (8.6)	2/1272 (0.2)	NG

Hansell et al. (1989)^24^	80.0	121	115/121 (95.0)	4/121 (3.3)	5/121 (4.1)	6/121 (5.0)	NG

Heinerman et al. (1989)^10^	56.3	190	189/190 (99.5)	1/190 (0.5)	4/190 (2.1)	0/190 (0.0)	NG

Ingoldby et al. (1989)^23^	79.7	186	172/186 (92.5)	4/186 (2.2)	9/186 (4.8)	3/186 (1.6)	3.7

Worthley (1989)^16^	73.0	50	44/50 (88.0)	7/50 (14.0)	12/50 (24.0)	0/50 (0.0)	3.0

Miller and Ferguson (1990)^15^	NG	58	52/58 (90.0)	6/58 (10.3)	6/58 (10.3)	1/58 (1.7)	3.6

**Total**	**56–83**	**3818**	**2210/2403 (92.0)**	**110/3306 (3.3)**	**312/3798 (8.2)**	**56/3818 (1.5)**	**3–6**

The short mean hospital stay of patients undergoing ES suggests that the added complication rates, mortality rate, and hospital stay from the subsequent cholecystectomy were not taken into account.

* The denominators include the total number of patients entered into the trial, after excluding those with suspected but absent CBD stones.

† The denominators include the number of patients with available data.

CBDI, common bile duct interventions; d, day(s); NG, not given; y, year(s).

**Table 3:** Uncontrolled Studies Published Before 1990 of the Outcomes of Cholecystectomy with Open Common Duct Exploration.

Author	Mean age y	Patients *n*	Stone Clearance *n* (%)*	Additional CBDI Needed (ES or Surg.) *n* (%)†	Complications *n* (%)†	Mortality *n* (%)	Length of Hospital Stay d (range)
Way et al. (1972)^29^	56.9	200	127/141 (90.1)	22/200 (11.0)	NG	5/200 (2.5)	NG

Stefanini et al. (1974)^28^	56.6	712	NG	NG	14/712 (2.0)	7/712 (1.1)	NG

Vellacott and Powel (1979)^35^	66	122	71/78 (92.2)	NG	11/122 (9.0)	9/122 (7.4)	16

Gaskill et al. (1982)^27^	56	71	64/71 (90.1)	NG	21/71 (29.6)	2/71 (2.8)	13

Antrum and Hall (1984)^34^	60.8	118	101/118 (85.6)	4/118 (3.4)	7/118 (5.9)	3/118 (2.5)	12

Crumplin et al. (1985)^33^	60.4	160	84/95 (88.4)	5/160 (3.1)	73/160 (45.6)	4/160 (2.5)	16.2

Rogers et al. (1985)^32^	60	100	46/55 (83.6)	3/100 (3.0)	NG	3/100 (3.0)	NG

Roukema et al (1986)^37^	<70	657	514/557 (92.3)	42/1007 (4.2)	203/1007 (20.2)	6/657 (0.9)	NG
	70–79	266				7/266 (2.6)	
	>80	84				8/84 (9.5)	

Neoptolemos et al. (1987)^9^	57.9	248	212/238 (89.1)	26/248 (10.5)	56/248 (22.6)	10/248 (4.0)	NG

Sheridan et al. (1987)^30^	57.4	257	163/200 (81.5)	22/257 (8.6)	118/257 (45.9)	5/257 (1.9)	15.6

Irvin and Arnstein (1988)^36^	<70	77	75/77 (97.4)	NG	5/77 (7.0)	0/77 (0.0)	10.4
	>70	69	66/69 (95.7)	NG	12/69 (17.4)	2/69 (2.9)	13.4

Miller et al. (1988)^14^	58.0	81	76/81 (93.8)	5/81 (6.2)	13/81 (16.0)	1/81 (1.2)	10.3

Heinerman et al. (1989)^10^	56.3	78	65/78 (83.3)	12/78 (15.4)	17/78 (21.8)	3/78 (3.8)	NG

McEntee et al. (1989)^31^	59.4	164	160/164 (97.6)	NG	NG	1/164 (0.6)	NG

Miller and Ferguson (1990)^15^	NG	42	33/42 (78.6)	9/42 (21.4)	10/42 (23.8)	1/42 (2.4)	10.4

Pappas et al. (1990)^26^	52.7	100	95/100 (95.0)	5/100 (5.0)	15/100 (15.0)	0/100 (0.0)	NG

**Total**	**52–>80**	**3606**	**1952/2164 (90.2)**	**155/2391 (6.5)**	**575/3142 (18.3)**	**77/3606 (2.1)**	**10.3–16**

* The denominators include the total number of patients entered into the trial, after excluding those with suspected but absent CBD stones.

† The denominators include the number of patients with available data.

CBDI, common bile duct interventions; d, day(s); NG, not given; y, year(s).

Table 4 summarizes the outcomes of the 26 randomized controlled trials that we could identify in the literature since Neoptolemos et al.^9^ published the first one in 1987, and Tables 5, 6, and 7 present their results in more detail. Before 2000, six of nine trials indicated that ES was associated with either a *lower* clearance of CBD stones or a *higher* mortality than open surgical CBD exploration. The remaining three studies indicated that ES and surgical treatment were equally effective (Table 5). In 2001–2010, five of eight trials showed that ES and laparoscopic surgery were equally safe and effective. In three studies, laparoscopic–ES rendezvous prevented post-ES pancreatitis and was associated with shorter hospital stays and less morbidity than ES alone (Table 6). Since 2011, four of nine trials found that laparoscopic surgery was preferred as it avoids cholangitis, papillary stenosis, or pancreatitis after ES, with less morbidity and earlier recovery. One of nine trials indicated that ES alone and laparoscopic surgery alone were equally safe and effective. The remaining four studies confirmed that laparoscopic–ES rendezvous prevents post-ES pancreatitis and is associated with shorter hospital stays (Table 7).

**Table 4:** Summary of the Outcomes of Randomized Controlled Trials Since 1980 Comparing Endoscopic Sphincterotomy (ES), Open Surgical Common Duct Exploration (Surg.), Laparoscopic Common Bile Duct Exploration (Lap.), and Laparo–ES Rendezvous (Lap.–ES).

Years	Intervention	Patients *n*	Stone Clearance *n* (%)*	Additional CBDI needed (ES or Surg.) *n* (%)†	Complications *n* (%)†	Mortality *n* (%)	Length of Hospital Stay d (range)
1980–2000	ES	472	301/358 (84.1)	70/472 (14.8)	73/472 (15.5)	10/472 (2.1)	3.5–17
	Surg.	313	201/220 (91.4)	37/265 (14.0)	60/313 (19.2)	4/313 (1.3)	6–22
	Lap.	173	130/149 (87.2)	29/149 (19.5)	28/173 (16.2)	1/173 (0.6)	1–6

2001–2010	ES	421	274/353 (77.6)	42/421 (9.8)	55/421 (13.1)	1/421 (0.2)	3–9
	Lap.	293	217/239 (90.8)	26/293 (8.9)	31/293 (10.6)	1/293 (0.3)	4–8
	Lap.–ES	165	123/131 (93.9)	9/165 (5.5)	8/165 (4.8)	0/165 (0)	3–5

2011–2023	ES	637	496/553 (89.7)	28/433 (6.5)	62/596 (10.4)	3/637 (0.5)	3–11
	Lap.	528	423/449 (94.2)	22/366 (6.0)	42/528 (8.0)	0/528 (0)	2–7
	Lap.–ES	220	155/167 (92.8)	3/79 (3.8)	8/178 (4.5)	1/220 (0.5)	1–7

* The denominators include the total number of patients entered into the trial, after excluding those with suspected but absent CBD stones.

† The denominators include the number of patients with available data.

CBDI, common bile duct interventions; d, day(s).

**Table 5:** Randomized Controlled Trials of the Outcomes of Endoscopic Sphincterotomy (ES), Open Surgical Duct Exploration (Surg.), and Laparoscopic Surgery (Lap.), 1980–2000.

Author	Intervention	Mean Age y (range or ±SD)	Patients *n*	Stone Clearance *n* (%)*	Additional CBDI needed (ES or Surg.) *n* (%)†	Minor and Major Complications *n* (%)†	Mortality *n* (%)	Length of Hospital Stay d (mean or mean range)	Author Conclusions
Neoptolemos et al. (1987)^9^	ES	61 (20–83)	55	50/55 (91.0)	2/55 (2.8)	18/55 (32.7)	2/55 (3.6)	9	No support for ES for CBD stones
	Surg.	59 (20–82)	59	54/59 (91.5)	0/59 (0.0)	13/59 (22.1)	1/59 (1.7)	11	

Stain et al. (1991)^38^	ES	48.4 (31–78)	26	17/26 (65.4)	1/26 (3.1)	4/26 (15.4)	0.0	5	No support for ES for CBD stones
	Surg.	42.4 (20–86)	26	16/17 (84.2)	1/17 (3.1)	8/26 (30.8)	0.0	6	

Stiegmann et al. (1992)^39^	ES	46.3±21.7	16	5/7 (71.4)	2/7 (28.6)	0 (0.0)	0.0	11.0	No advantage to treating CBD stones with ES
	Surg.	38.1±14.8	18	6/7 (85.7)	Not given	3/18 (16.7)	0.0	9.2	

Hammarström et al. (1995)^40^	ES	75.0 (56–85)	39	35/39 (89.7)	7/39 (17.9)	7/39 (17.9)	0.0	13‡	ES and Surg. for CBD stones are equally effective
	Surg.	73.5 (56–85)	41	37/41 (90.2)	6/41 (14.6)	9/41 (22.0)	0.0	16‡	

Kapoor et al. (1996)^42^	ES	42 (20–60)	13	11/13 (85)	2/13 (15)	5/13 (38.5)	0 (0.0)	10.6	No support for ES in low-risk patients with CBD
	Surg.	46 (24–75)	16	13/15 (87)	2/15 (13)	5/16 (31.3)	0 (0.0)	11.3	

Targarona et al. (1996)^41^	ES	79	50	NG	2/50 (4.0)	8/50 (16)	3/50 (6.0)	17	In elderly or high-risk patients, surgery is preferable to ES
	Surg.	80	48	NG	1/48 (2.1)	11/48 (23)	2/48 (4.0)	22	

Rhodes et al. (1998)^44^	ES	68 (28–84)	40	37/40 (92.5)	10/40 (25.0)	6/40 (15.0)	0 (0.0)	3.5‡	Lap. is as effective as ES in clearing CBD stones
	Lap.	62 (24–83)	40	40/40 (100)	10/40 (25.0)	7/40 (17.5)	0 (0.0)	1‡	

Suc et al. (1998)^43^	ES	66.8±17.5	97	64/80 (80.0)	28/97 (28.9)	11/97 (11.3)	3/97 (3.1)	15.3	High risk of added procedures after ES precludes its use for treating CBD stones
	Surg.	66.7±18.1	105	75/81 (92.6)	8/105 (7.6)	13/105 (12.3)	1/105 (1.0)	17.5	

Cuschieri et al. (1999)^45^	ES	18–89	136	82/98 (83.7)	16/98 (16.3)	17/133 (12.8)	2/136 (1.5)	9‡	Similar outcomes for ES and Lap., shorter hospital stay with Lap. ES to be confined to high-risk patients
	Lap.	19–88	133	90/109 (82.6)	19/109 (17.4)	21/133 (15.8)	1/133 (0.8)	6‡	

**Total**	**ES**		**472**	**301/358 (84.1)**	**70/472 (14.8)**	**73/472 (15.5)**	**10/472 (2.1)**	**3.5–17**	
	**Surg.**		**313**	**201/220 (91.4)**	**37/265 (14.0)**	**60/313 (19.2)**	**4/313 (1.3)**	**6–22**	
	**Lap.**		**173**	**130/149 (87.2)**	**29/149 (19.5)**	**28/173 (16.2)**	**1/173 (0.6)**	**1–6**	

* The denominators include the total number of patients entered into the trial, after excluding those with suspected but absent CBD stones.

† The denominators include the number of patients with available data.

‡ Median.

CBDI, common bile duct interventions; d, day(s); NG, not given; y, year(s).

**Table 6:** Randomized Controlled Trials Comparing Outcomes of Endoscopic Sphincterotomy (ES) Laparoscopic Common Duct Exploration (Lap.), and Laparo-Endoscopic Rendezvous Technique (Lap.-ES), 2001–2010.

Author	Intervention	Mean Age y (range or SD)	Pts. *n*	Stone Clearance *n* (%)*	Additional CBDI needed (ES or Surg.) *n* (%)†	Minor and Major Complications *n* (%)†	Mortality *n* (%)	Length of Hospital Stay d (mean or mean range)	Author Conclusions
Sgourakis and Karaliotas (2002)^46^	ES	46–89	42	27/32 (84.3)	5/42 (11.9)	6/42 (14.3)	1/42 (2.4)	9.0	There is a need for further randomized trials
	Lap.	43–88	36	24/28 (85.7)	4/36 (11.1)	5/36 (13.9)	1/36 (2.8)	7.4	

Hong et al. (2006)^47^	ES	Not given	93	85/93 (91.4)	8/93 (8.6)	9/93 (9.4)	0 (0.0)	4.3	ES and Lap. are safe and effective treatments for CBD stones
	Lap.	48 (15–82)	141	126/141 (89.4)	15/141 (10.6)	8/141 (5.6)	0 (0.0)	4.7	

Lella et al. (2006)^52^	ES	Not given (<60)	60	58/60 (96.7)	0/60 (0.0)	8/60 (13.3)	0 (0.0)	6	Lap.-ES prevents post-ES pancreatitis in patients at risk for this complication
	Lap.-ES	Not given (<60)	60	59/60 (98.3)	2/60 (3.3)	2/60 (3.3)	0 (0.0)	3	

Morino et al. (2006)^4^	ES	63.1 (25–83)	45	9/45 (80.0)	17/45 (37.8)	3/45 (6.6)	0 (0.0)	8.0	Lap.-ES carries a higher rate of CBD stones clearance and shorter hospital stays than ES
	Lap.-ES	56.6 (22–82)	46	44/46 (95.6)	3/46 (6.5)	3/46 (6.5)	0 (0.0)	4.3	

Rábago et al. (2006)^51^	ES	Not given (<80)	64	28/31 (90.5)	7/64 (10.9)	12/64 (18.8)	0 (0.0)	8	Lap.-ES had less morbidity and a shorter hospital stay than ES
	Lap.-ES	Not given (<80)	59	20/25 (80.0)	4/59 (6.8)	3/59 (5.1)	0 (0.0)	5	

Noble et al. (2009)^48^	ES	74.3 (70–79)	47	26/46 (56.5)	2/47 (4.3)	8/47 (17.0)	0 (0.0)	3	No difference in hospital stay, complications, or conversion. Lap. was more effective and avoided unnecessary procedures
	Lap.	75.9 (70–81)	44	38/38 (100)	4/44 (9.1)	8/44 (18.2)	0 (0.0)	5	

Bansal et al. (2010)^50^	ES	39.1 (23–64)	15	11/15 (73.3)	2/15 (13.3)	4/15 (26.7)	0 (0.0)	4.0	Equivalent morbidity and hospital stay. Lap. carried a smaller number of procedures
	Lap.	47.1 (34–72)	15	14/15 (93.3)	1/15 (6.7)	4/15 (26.7)	0 (0.0)	4.2	

Rogers et al. (2010)^49^	ES	44.6±1.9	55	30/31 (98)	1/55 (1.8)	5/55 (9.1)	0 (0.0)	6.6	ES and Lap. are highly effective in treating CBD stones. Hospital stay was shorter for Lap.
	Lap.	39.9±1.9	57	15/17 (88)	2/57 (3.5)	6/57 (10.5)	0 (0.0)	5.3	

**Total**	**ES**		**421**	**274/353 (77.6)**	**42/421 (9.8)**	**55/421 (13/1)**	**1/421 (0.2)**	**3–9**	
	**Lap.**		**293**	**217/239 (90.8)**	**26/293 (8.9)**	**31/293 (10.6)**	**1/293 (0.3)**	**4.2–7.4**	
	**Lap.-ES**		**165**	**123/131 (93.9)**	**9/165 (5.5)**	**8/165 (4.8)**	**0/165 (0.0)**	**3–5**	

* The denominators include the total number of patients entered into the trial, after excluding those with suspected but absent CBD stones.

† The denominators include the number of patients with available data.

CBDI, common bile duct interventions; d, day(s); Pts., patients; y, year(s).

**Table 7:** Randomized Controlled Trials Comparing Outcomes of Endoscopic Sphincterotomy (ES), Laparoscopic Common Duct Exploration (Lap.), and Laparo-Endoscopic Rendezvous Technique (Lap.-ES), 2011–2023.

Author	Intervention	Mean Age y (range or SD)	Patients *n*	Stone Clearance *n* (%)*	Additional CBDI Needed (ES or Surg.) *n* (%)†	Complications *n* (%)†	Mortality *n* (%)	Length of Hospital Stay d (mean or mean range)	Author Conclusions
ElGeidie et al. (2011)^53^	ES	29.2 (20–67)	111	98/107 (91.6)	7/111 (6.3)	10/111 (9.3)	0 (0.0)	3.1	ES and Lap. safe and effective treatments for CBD stones
	Lap.	32.5 (19–64)	115	104/110 (94.5)	9/115 (7.8)	8/115 (7.0)	0 (0.0)	2.2	

Ferulano et al. (2011)^54^	ES	55±15	62	37/39 (95)	Not given	6/62 (9.7)	0 (0.0)	3.5	Lap. avoids cholangitis, papillary stenosis, or pancreatitis after ES
	Lap.	53±13	62	42/45 (93)	Not given	4/62 (6.5)	0 (0.0)	7.1	

Tzovaras et al. (2012)^59^	ES	69 (25–85)	49	39/49 (79.6)	1/49 (2.0)	6/49 (12.2)	0 (0.0)	5.5	Lap.-ES carries less hospital stay and lower amylase levels than ES
	Lap.-ES	66 (22–87)	50	44/50 (88.0)	2/50 (4.0)	7/50 (14)	1/50 (2.0)	4	

Koc et al. (2013)^55^	ES	54.9±17.9	54	51/54 (94.4)	1/54 (1.9)	6/54 (11.1)	0 (0.0)	6	Lap. has less morbidity and allows earlier recovery than ES
	Lap.	51.5±16.6	57	55/57 (96.5)	0/57 (0.0)	4/57 (7.0)	0 (0.0)	3	

Bansal et al. (2014)^56^	ES	45.1±15.1	84	73/83 (88.0)	16/84 (19.1)	19/84 (22.6)	3/84 (3.7)	5.3	Lap. has shorter hospital stays and needs fewer procedures
	Lap.	43.0±13.7	84	77/84 (91.7)	10/84 (11.9)	20/84 (23.8)	0 (0.0)	4.6	

Ding et al. (2014)^57^	ES	57.5±6.3	111	105/111 (94.6)	1/111 (0.9)	6/111 (5.4)	0 (0.0)	NG	Lap. is the treatment of choice for patients with CBD stones
	Lap.	58.4±7.2	110	103/110 (93.6)	3/110 (2.7)	4/110 (3.6)	0 (0.0)	NG	

Sahoo et al. (2014)^60^	ES	48 (21–75)	41	29/41 (70.7)	NG	NG	0 (0.0)	10.9	Lap.-ES reduces pancreatitis, hospital stays, and interventions
	Lap.-ES	48 (21–75)	42	38/42 (90.5)	NG	NG	0 (0.0)	6.8	

Barreras González et al. (2016)^61^	ES	57.7 (20–84)	101	42/45 (93.3)	Not given	6/101 (5.9)	0 (0.0)	3.1	Lap.-ES shows a higher rate of CBD stone clearance, a shorter hospital stay, and lower morbidity
	Lap.	56.3 (22–87)	100	42/43 (97.7)	Not given	2/100 (2.0)	0 (0.0)	2.1	
	Lap.-ES	58.4 (23–87)	99	45/46 (97.8)	Not given	0/99 (0.0)	0 (0.0)	1.2	

Lv et al. (2016)^58^	ES	63.5±12.4	24	22/24 (91.7)	2/24 (8.3)	3/24 (12.5)	0 (0.0)	10.9	Lap.-ES resulted in a shorter hospital stay
	Lap.-ES	61.3±14.5	29	28/29 (96.6)	1/29 (3.4)	1/29 (3.4)	0 (0.0)	6.7	

**Total**	**ES**		**637**	**496/553 (89.7)**	**28/433 (6.5)**	**62/596 (10.4)**	**3/637 (0.5)**	**3.1–10.9**	
	**Lap.**		**528**	**423/449 (94.2)**	**22/366 (6.0)**	**42/528 (8.0)**	**0/528 (0.0)**	**2.1–7.1**	
	**Lap.-ES**		**220**	**155/167 (92.8)**	**3/79 (3.8)**	**8/178 (4.5)**	**1/220 (0.5)**	**1.2–6.8**	

* The denominators include the total number of patients entered into the trial, after excluding those with suspected but absent CBD stones.

† The denominators include the number of patients with available data.

CBDI, common bile duct interventions; d, day(s); NG, not given; y, year(s).

## CONCLUSIONS

Three main findings emerge from the present review: first, as expected, since 1990, there has been a uniform decline in mortality, complications, and the need for added interventions in the treatment of choledocholithiasis by ES and laparoscopic CBD exploration. Second, since 2001, the differences in mortality (0% and 0.5%) and stone clearance (89.7% and 94.2%) between ES and laparoscopic CBD exploration were negligible. Third, laparoscopic–ES rendezvous was associated with fewer additional interventions, fewer complications, and shorter hospital stays.

Therefore, the combined approach (laparoscopic–ES rendezvous) seems to be the preferred one for the treatment of patients with choledocholithiasis. Unfortunately, this requires a high degree of collaboration between departments in those environments where this procedure is done by different services, and therefore cannot be performed in environments with low resources.

The first limitation of the presented review is its failure to assess the quality of the evidence and the risk of bias in the individual studies. Second, since the presented review is not systematic, it is possible that we missed relevant studies. Still, the consistency of our findings over the last two decades supports their validity. Therefore, we conclude that, *given experience and facilities, the laparoscopic–ES rendezvous is preferable over ES alone or laparoscopic surgery alone*. The combined approach obviates the need to cut the papilla, thereby preventing hemorrhage and perforation, and improves the selective cannulation of the CBD, thereby preventing inadvertent cannulation of the pancreatic duct with the injection of contrast media into it and pancreatitis.^62^

Should ES alone be confined only to patients with retained CBD stones after cholecystectomy, and to high-risk patients with CBD stones and intact gallbladders? We know of no comparative studies of the outcome of ES alone and the rendezvous technique by patients’ age. Therefore, we believe that the role of the laparoscopic–ES rendezvous procedure in the treatment of high-risk patients with CBD stones remains uncertain. It should be the subject of future trials that will probably address also cost.

## Abbreviations

CBD: common bile duct
ES: endoscopic sphincterotomy
Surg.: surgical common duct exploration
